# Social, Economic, and health risks among people who use Methamphetamine: Comparing three patterns of opioid Co-Use

**DOI:** 10.1016/j.abrep.2025.100660

**Published:** 2025-12-19

**Authors:** Kimberly Page, Mia Rae Kirk, Tristin Garcia, Haley Etchart, Benjamin Chase, Robert W. Harding, Jess Anderson, May McCarthy, Phillip Fiuty, Kathleen Reich, Kelly Mytinger, Olufemi Erinoso, Karla D. Wagner

**Affiliations:** a Division of Epidemiology, Biostatistics, and Preventive Medicine, Dept. of Internal Medicine, University of New Mexico Health Sciences Center, Albuquerque, NM, United States; b Department of Health Behavior, Policy, and Administration Sciences, School of Public Health, University of Nevada, Reno, Reno, NV, United States; c Adulterant Checking Program, Opioid Response Harm Reduction Section, NM Health, Las Cruces, NM, United States; d Trac-B Exchange, Las Vegas, NV, United States; e The Mountain Center, Española, NM, United States

**Keywords:** Methamphetamine, Opioids, Polydrug use, Goofballs

## Abstract

**Objectives:**

To examine how persons using different methamphetamine-opioid combinations differ with respect to social, risk, and health characteristics, and to identify patterns of vulnerability across simultaneous, sequential, and independent use.

**Methods:**

414 people who use illicit drugs were surveyed, 384 of whom reported polydrug use involving methamphetamine, in Nevada and New Mexico between June 2022 and August 2023. Participants were classified into three mutually exclusive groups based on self-reported methamphetamine and opioid use patterns: simultaneous use (deliberately using both drugs together), sequential use (using both drugs but not deliberately using simultaneously), and independent use (using methamphetamine alone or with non-opioid drugs). Prevalence ratios were calculated to examine associations between use patterns and social, economic, health, and drug use characteristics.

**Results:**

We identified three distinct use patterns: simultaneous (53.1%), sequential (17.7%), and independent (29.2%). Individuals in these risk groups exhibited differential social, economic, carceral, and health-related risk: those engaged in simultaneous showed higher vulnerability relative to people who use independently and sequentially. Healthcare engagement was associated with lower simultaneous use.

**Conclusions:**

These findings suggest the need to support development of strategies to support transition from higher to lower-risk drug use patterns.

## Highlights

• Over half of participants reported simultaneous methamphetamine-opioid use.
• People with simultaneous use had highest rates of homelessness, trauma, and incarceration.
• Healthcare engagement associated with lower risk of simultaneous drug use.

## 1 Introduction

Co-use of methamphetamine with opiates, opioid analogues, and prescription opioids (hereafter referred to collectively as opioids) and associated morbidity and mortality are increasing in the United States, with the greatest increases occurring between 2019 and 2021 (Sauda et al., 2024). Psychostimulant-involved overdose events in emergency departments increased 11 % yearly from 2010 to 2015, and 19.1 % from 2016 (11.4/100,000 age adjusted population (aap)) to 2017 (13.6/100,000 app), with over a third (36 %) involving multiple drugs (mostly opioids) (Vivolo-Kantor et al., 2020). Treatment admissions involving methamphetamine have risen (Jones et al., 2020) as have overdose deaths involving psychostimulants (Hoopsick & Andrew Yockey, 2023).

These increases, in both rural and urban areas (Ellis et al., 2021, Jones et al., 2022), have been characterized as a ‘resurgent’ stimulant epidemic, which is made more complex by high rates of polydrug use of methamphetamine and opioids (Ciccarone, 2021, Compton et al., 2021, Ellis et al., 2021, Hoopsick and Andrew Yockey, 2023, Jones et al., 2022).

Polydrug use is the purposeful consumption of more than one substance. However, use patterns are not homogeneous, and polydrug use includes use of multiple drugs simultaneously, sequentially, or in other patterns (Bunting et al., 2023). Reasons for intentionally mixing methamphetamine and opioids are varied and include using one drug to mitigate the negative effects of the other (e.g., using opioids to treat the negative effects of coming off a methamphetamine binge), enhancing or prolonging a high, or to experience different drug effects (Rhed et al., 2022). The well-documented simultaneous use (by injection or ingestion) of methamphetamine and opioids is known as a “goofball” (Glick et al., 2021).

**Fig. 1:**
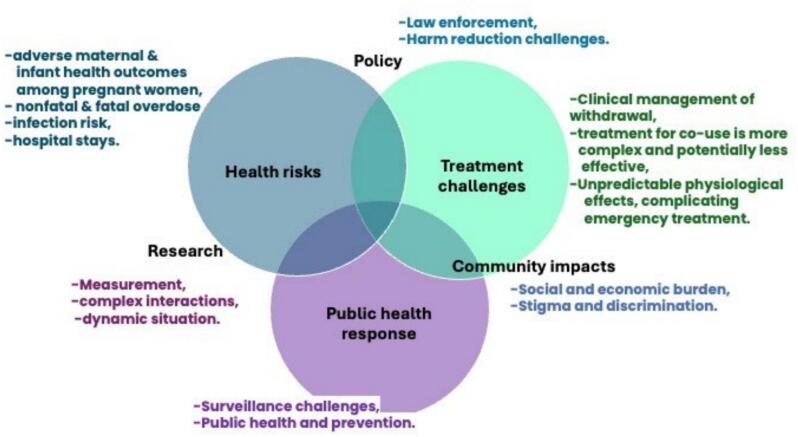
Schematic illustrating complexities of co-use of methamphetamine and opioids on treatment, public health and harm reduction responses and community impacts.

Co-use of methamphetamine with opioids introduces health risks, and complexities in treatment, public health and harm reduction responses, and community impacts illustrated in Fig. 1 (Jones et al., 2022). Health risks include increased risk of infection (Wagner et al., 2021), hospital stays (Shearer et al., 2024b), nonfatal and fatal overdose, and adverse maternal and infant health outcomes among pregnant women (Palis et al., 2022, Pippard et al., 2024). Combining stimulants with opioids can lead to unpredictable physiological effects complicating diagnosis and emergency treatment (Glidden et al., 2023). Clinical management of withdrawal is complex and comprehensive treatment for co-use is less effective (Shearer et al., 2024a). There is a lower probability of receiving treatment for MOUD (Frost et al., 2019) and higher risk of relapse amongst people in treatment (Krawczyk et al., 2021) when they engage in opioid and stimulant co-use. The additive or synergistic pharmacological effects of combining these two substances at a neurochemical level are not fully understood (Bedard et al., 2023), complicating the development of effective treatment strategies. Whether intentional or unintentional (Nguyen et al., 2024), rising prevalence of co-use of methamphetamine and opioids and associated negative health impacts point to the need for in-depth research to develop and implement effective public health responses, as well as clinical and behavioral treatments targeting polydrug use.

Here we report findings from the AMPED 2.0 project, a multi-site study in New Mexico (NM) and Nevada (NV) designed to examine patterns of methamphetamine use in the context of an entrenched and ongoing opioid use epidemic in rural and urban areas of the Mountain West. We examine three mutually exclusive groups who report polydrug use involving methamphetamine: 1) those who report simultaneous use of methamphetamine and opioids (i.e., goofballs); 2) those who report sequential use of methamphetamine and opioids; and 3) those who report use of methamphetamine and other non-opioid drugs. We assess associations between geographic, demographic, socioeconomic, physical and mental health, and drug use exposures. The goal was to determine how people reporting different methamphetamine polydrug use patterns differ with respect to social, health, and contextual risk factors. Given previous reports of higher risk among people who use goofballs relative to those who do not report mixing drugs (Glick et al., 2021) as well as more unmet social and harm reduction service needs (Sun et al., 2024), we hypothesized that people there may be further differences in social, health, and contextual risk circumstances that have not been fully explored in people who either endorse using methamphetamine and opioids sequentially, or with other non-opioid substances.

## 2 Methods

*Ethics.* The study protocol was reviewed and approved by the University of Nevada Reno Institutional Review Board (UNR IRB) which served as the single IRB for the study. University of New Mexico Health Sciences Center relied on the UNR IRB for this multisite research. A Certificate of Confidentiality was obtained from the Centers of Disease Control and Prevention to add further protections for participant confidentiality.

*Study setting:* The AMPED 2.0 study was conducted in two metropolitan urban and semi-urban areas in two Mountain West states. Participants were recruited in Reno and Las Vegas, NV, and in Espanola and Albuquerque, NM. Data were collected between September 2022 and August 2023. Recruitment was conducted using street and agency-based outreach. Agency sites included community-based harm reduction organizations providing syringe, social, and some clinical services for people who use drugs (PWUD). The NV sites included *Trac-B Impact Exchange* in Las Vegas and *Change Point* in Reno. The NM sites included *The Mountain Center* in Espanola and the *Transgender Resource Center* in Albuquerque. People from these sites participated in conceiving of, planning, and implementing the study.

*Participants:* Inclusion criteria were being an adult 18 years of age or older, who reported illicit substance use in the past three months (excluding only alcohol, tobacco, or cannabis use), and spoke English or Spanish. The study employed both convenience (word of mouth, and face to face) and incentivized snowball recruitment methods wherein participants were given the option to recruit up to three people who might qualify for the study. Participants who agreed to recruit were provided referral coupons with unique codes to give to the person they referred. Referring participants were compensated $10 when coupons were redeemed by eligible individuals (for up to $30). Participants were compensated $40 for their participation. People who were unable to comprehend or provide informed consent for participation as judged by the research staff, based on consent recall questions, were excluded.

*Data Collection:* Cross-sectional surveys were administered by trained study staff at the four study sites. Staff were trained in the principles of rigorous and ethical data collection and met with participants in private/semi-private locations for private conversations. Data collection locations included public libraries, research van, harm reduction resource centers, syringe service programs, health clinics, and a dedicated field data collection site. Eligibility screening was conducted using a brief anonymous questionnaire administered by research staff and entered into a Research Electronic Data Capture (REDCap) system. Participants who provided written informed consent were enrolled and compensated prior to beginning the survey.

*Measures:* The interview (which generally took 45 to 60 min to complete) included queries regarding sociodemographic factors, mental/behavioral health, physical health, recent access to healthcare, drug use, and experiences seeking treatment for substance use disorders, including over their lifetime and recently (last 3 months).

Sociodemographic factors included age, gender identity, race/ethnicity, educational attainment, employment status, housing status in the past 3 months, household financial situation (categorized as lives comfortably, meets basic expenses with a little leftover, or doesn't have enough to meet basic expenses), and food insecurity. Food insecurity was assessed using two questions adapted from the U.S. Household Food Security Survey Module asking how often in the past 12 months participants worried that food they bought didn't last and they didn't have money to get more, and how often they worried they would run out of food before getting money to buy more (response options: often, sometimes, never). History of incarceration (ever and in the past 3 months) was also assessed. Mental and behavioral health measures included self-reported mental health concerns (participants were asked if they had any mental health concerns and could select from a list including depression, anxiety, PTSD, bipolar disorder, schizophrenia, and other), whether they had ever been diagnosed by a health provider with a mental health condition, current use of prescribed medications for mental health, use of non-prescribed medications for mental health, engagement in therapy or counseling for mental health, and whether they were prescribed stimulants during childhood. Trauma history was assessed by asking about lifetime trauma or stress, recent trauma or stress (past 3 months), and childhood trauma. Physical health and healthcare access measures included current health insurance status, recent healthcare provider visits (past 3 months), and diagnoses by healthcare providers of hepatitis C virus (HCV) infection, sexually transmitted infections, and skin infections (past 3 months). Drug use behaviors included queries about substances previously and currently using including use for various substances (alcohol, heroin, fentanyl, methamphetamine, prescription opioids, prescription amphetamines), history of injection drug use (ever and in the past 3 months), sharing of previously used syringes (ever and in the past 3 months), and transitions in drug use (e.g., previously used heroin but now use fentanyl, previously used heroin but now use methamphetamine). Participants were asked if they “*deliberately take two or more different kinds of drugs together simultaneously* and specifically if they used methamphetamine with ≥ 1 opioid (heroin, fentanyl, and or prescription opioids). Treatment experiences included whether participants had ever received treatment for substance use disorder, recent or current treatment for substance use disorder (past 3 months), whether they had ever been prescribed medications for opioid use disorder (MOUD), and recent or current MOUD treatment (past 3 months).

*Analyses:* Three mutually exclusive groups were created based on reported methamphetamine and opioid use. Participants who endorsed *deliberately taking two or more different kinds of drugs together simultaneously* and reported using methamphetamine with ≥ 1 opioid (heroin, fentanyl, and/or prescription opioids) were classified as using “Simultaneously”. Participants who endorsed using methamphetamine and opioids but did not deliberately take them simultaneously were classified as using in a “Sequential” pattern, and those who reported methamphetamine use alone or use with *any non-opioid drug* were classified as using in an “Independent” pattern. Descriptive statistics including means and medians and statistical dispersion were tabulated for demographic and risk exposure measures in the sample overall, and in the three groups. Bivariate analyses (chi-square or Fisher's exact for tables with at least one cell size ≤ 5), prevalence ratios, and associated 95 % Confidence Intervals (95 % CI) were used to examine associations between geographic, demographic, socioeconomic (e.g., food, clothing, and housing needs), physical and mental health factors, and drug use behaviors among the three groups. Adjusted prevalence ratios (aPRs) were generated to compare the Simultaneous group to the Sequential and Independent groups, using modified Poisson regression models with robust error variance (Barros & Hirakata, 2003). This methodological approach is consistent with presenting prevalence ratios and estimating adjusted prevalence ratios and relative risk in cross sectional studies. It provides more interpretable and accurate estimates of association and excess risk when outcomes are common, and eliminates risk of substantially overestimating relative risk when outcomes are not rare (Cummings, 2009, Ranganathan et al., 2015).

Variables included in the multivariable models were those found to be significant in bivariate analyses at p ≤ 0.10, known potential confounders (e.g., age, sex, location), and those hypothesized a priori to be associated (e.g., a history of injection drug use, being previously prescribed stimulants, unstable housing, and poor socioeconomic conditions). In these models, use pattern (simultaneous vs. sequential, or simultaneous vs. independent) was the outcome variable, and demographic, socioeconomic, health, and drug use factors were predictor variables. We used a mixed selection approach and selected a final parsimonious model for each comparison after comparing full and nested models and considering clinical and social significance. Analyses were conducted with SAS 9.4 (SAS Institute, Cary, NC) and STATA *[version 18.5]* (College Station, TX).

## 3 Results

A total of 570 people were screened of whom 545 (95.6 %) were eligible, and 423 (77.6 %) enrolled. Among those enrolled, 420 (99.3 %) started the survey and 414, (97.9 %) completed the survey. The primary reason for not enrolling was failure to make the scheduled interview appointment. Among all 414 respondents, 384 (92.3 %) reported polydrug use involving methamphetamine. The median age of all participants was 39 (Interquartile Range (IQR) 33, 49.5) years, with NM participants slightly younger than NV participants (median 38 vs. 43, respectively). Just under half (43 %) identified as female. The majority of Hispanic (75.6 %) and American Indian/Alaskan Native (AI/AN) (9.4 %) participants were sampled in NM compared to 23.3 % and 5.9 %, respectively, in NV. The NM sample also had a higher proportion of participants with less than a high school education (30.9 %) relative to NV (15 %). Overall, 69 % reported experiencing homelessness in the past 3 months, and 60.7 % endorsed financial insecurity. A majority (91.7 %) reported currently having health insurance and almost half (45.8 %) had recently seen a health care provider. Almost all (90.4 %) participants reported at least one mental health concern, and 14.3 % (n = 55) reported currently taking prescribed medication for mental health. Table 1 shows prevalence of demographic, socioeconomic, health and healthcare characteristics, substance use, and substance use disorder treatment among all participants.

**Table 1:** Participant characteristics and prevalence of polydrug use involving methamphetamine, stratified by use pattern, and bivariate associations with demographic, socioeconomic, physical and mental health, and drug use behaviors.

	Prev of characteristic		Simultaneous (Meth & Opioid (Goofball))		Sequential (Meth & Opioid)		Prevalence ratio: Simultaneous use vs. Sequential use		Independent (Meth & No Opioid)		Prevalence ratio: Simultaneous use vs. Independent use	
Demographic characteristic	N = 384		n = 204 (53.13 %)		N = 68 (17.71 %)				N = 112 (29.17 %)			
	N	Column %	N	%	N	%	PR	95 %CI	N	%	PR	95 %CI
State												
Nevada	193	50.26 %	77	39.90	31	16.06	1		85	44.04	**1**	
New Mexico	191	49.74 %	127	66.49	37	19.37	1.09	0.94, 1.26	27	14.14	**1.74**	**1.45, 2.07**
Gender												
Male	211	54.95 %	117	55.45	37	17.54	1.02	0.89, 1.17	57	27.01	1.08	0.91, 1.27
Female	165	42.97 %	85	51.52	29	17.58	1		51	30.91	1	
Trans/Binary	8	2.08 %	2	25.00	2	25.00	0.67	0.25, 1.80	4	50.00	0.52	0.17, 1.67
Education												
< HS	88	22.92 %	50	56.82	17	19.32	1.01	0.85, 1.20	21	23.86	1.12	0.92, 1.36
HS or GED	125	32.55 %	66	52.80	20	16.00	1.04	0.89, 1.22	39	31.2	1.00	0.82, 1.21
>= College	171	44.53 %	88	51.46	31	18.13	1		52	30.41	1	
Socioeconomic characteristic												
Employment status												
Unemployed	280	73.11 %	156	55.71	49	17.50	1.01	0.84, 1.21	75	26.79	1.23	0.97, 1.57
Full/part time employed	73	19.06 %	34	46.58	11	15.07	1		28	38.36	**1**	
Unable to work	30	13.51 %	14	46.67	7	23.33	0.88	0.62, 1.25	9	30.00	1.11	0.75, 1.65
Homeless in the past 3 months												
No	119	30.99 %	48	40.34	33	27.73	**1**		38	31.94		
Yes	265	69.01 %	156	58.87	35	13.21	**1.38**	**1.14, 1.67**	74	27.92	**1.22**	**0.99, 1.50**
Household financial situation												
Lives comfortably	54	14.10 %	18	33.33	12	22.22	**1**		24	44.44	**1**	
Meets basic expenses with a little leftover	96	25.07 %	50	52.08	22	22.92	1.16	0.83, 1.61	24	25.00	**1.58**	**1.07, 2.31**
Don't have enough to meet basic expenses	233	60.84 %	136	58.37	34	14.59	1.33	0.99, 1.80	63	27.04	**1.59**	**1.11, 2.29**
How often food you bought didn’t last and [they] had no money to buy more (past 12 mo)												
Often	158	41.25 %	94	59.49	22	13.92	**1.31**	**1.07, 1.61**	42	26.58	1.27	1.0, 1.6
Sometimes	122	31.85 %	68	55.74	19	15.57	**1.27**	**1.02, 1.57**	35	28.69	1.21	0.95, 1.55
Never	103	26.89 %	42	40.78	26	25.24	**1**		35	33.98	**1**	
History of incarceration												
Ever No	41	10.68 %	17	41.46	7	17.07	1		17	41.46	1	
Yes	343	89.32 %	187	54.52	61	17.78	1.06	0.82, 1.39	95	27.70	1.32	0.94, 1.88
Past 3 Mo. No	311	80.99 %	154	49.52	59	18.97	**1**		98	31.51	1	
Yes	73	19.01 %	50	66.67	9	12.33	**1.17**	**1.02, 1.34**	14	19.18	**1.28**	**1.09, 1.5**
Health and health care factors												
Has health insurance												
No	28	7.37 %	15	53.57	4	14.29	1		9	32.14	1	
Yes	352	92.63 %	187	53.13	64	18.18	0.94	0.74, 1.20	101	28.69	1.04	0.75, 1.43
Recent health provider visit												
No	208	54.17 %	125	60.10	36	17.31	1		47	22.60	**1**	
Yes	176	45.83 %	79	44.89	32	18.18	0.92	0.79, 1.06	65	36.93	**0.75**	**0.63, 0.90**
Ever been diagnosed by health provider with any mental health problem												
No	367	95.57 %	197	53.68	61	16.62	1		109	29.7	1	
Yes	17	4.43 %	7	41.18	7	41.18	0.65	0.39, 1.11	3	17.65	1.09	0.72, 1.65
Any mental health concern												
No	37	9.64 %	11	29.73	13	35.14	**1**		13	35.14	1	
Yes	347	90.36 %	193	55.62	55	15.85	**1.7**	**1.09, 2.64**	99	28.53	1.44	0.93, 2.25
Currently taking any prescribed medication for mental health												
No	329	85.68 %	185	56.23	52	15.81	**1**		**92**	**27.96**	1	
Yes	55	14.32 %	19	34.55	16	29.09	**0.70**	**0.51, 0.95**	**20**	**36.36**	0.73	0.52, 1.02
Currently taking any non-prescribed medication for mental health												
No	368	95.83 %	192	52.17	65	17.66	1		111	30.16	**1**	
Yes	16	4.17 %	12	75.00	3	18.75	1.07	0.82, 1.39	1	6.25	**1.46**	**1.22, 1.74**
Ever been in therapy/counseling for mental health												
No	319	83.07 %	177	55.49	55	17.24	1		87	27.27	1	
Yes	65	16.93 %	27	41.54	13	20.00	0.88	0.71, 1.11	25	38.46	0.77	0.59, 1.02
Prescribed stimulants in childhood												
No	308	80.21 %	158	51.3	59	19.16	**1**		91	29.55	1	
Yes	76	19.79 %	46	60.53	9	11.84	1.15	1.0, 1.32	21	27.63	1.08	0.90, 1.30
History of lifetime trauma/stress												
No	54	14.06 %	19	35.19	23	42.59	**1**		12	22.22	1	
Yes	330	85.94 %	185	56.06	45	13.64	**1.78**	**1.27, 2.50**	100	30.30	1.06	0.79, 1.42
Recent (past 3 mo) trauma/stress												
No	130	33.85 %	56	43.08	36	27.69	**1**		35	29.23	1	
Yes	254	66.15 %	148	58.27	32	12.60	**1.35**	**1.13, 1.61**	74	29.13	1.08	0.90, 1.31
Childhood trauma												
No	66	17.19 %	26	39.39	27	40.91	**1**		13	19.70	1	
Yes	318	82.81 %	178	55.97	41	12.89	**1.66**	**1.25, 2.2**	99	31.13	0.96	0.76, 1.22
Diagnosed with active HCV infection by health provider ever												
No	253	66.06 %	124	49.01	44	17.39	1		**85**	**33.60**	**1**	
Yes	130	33.94 %	80	61.54	24	18.46	1.04	0.91, 1.2	**26**	**20.00**	**1.27**	**1.09, 1.49**
Diagnosed with sexually transmitted infection ever												
No	286	69.08 %	150	**52.45**	49	18.15	1		71	26.30	1	
Yes	128	30.92 %	54	**42.19**	19	16.67	0.98	0.84,1.15	41	35.97	0.84	0.69, 1.02
Diagnosed with skin infection recently (past 3 mo)												
No	273	83.23 %	143	52.38	50	18.32	1		80	29.3	1	
Yes	55	16.77 %	38	69.09	9	16.36	1.09	0.93, 1.28	8	14.55	**1.29**	**1.09, 1.52**
Drug Use												
Previously used heroin but now use fentanyl (N = 255)												
No	80	31.37 %	46	57.5	22	27.5	**1**		**12**	**15**	**1**	
Yes	175	68.63 %	145	82.86	30	17.14	**1.22**	**1.03, 1.46**	**0**	**0**	**1.26**	**1.11, 1.44**
Previously used heroin but now methamphetamine (N = 271)												
No	184	66.67 %	127	69.02	33	17.93	1		24	13.04	1	
Yes	87	31.52 %	58	66.67	17	19.54	0.97	0.84, 1.13	12	13.79	0.99	0.87, 1.12
Ever injected drugs												
No	77	20.05 %	24	31.17	10	12.99	1		43	55.84	**1**	
Yes	307	79.95 %	180	58.63	58	18.89	1.07	0.85, 1.35	69	22.48	**2.02**	**1.45, 2.81**
Ever shared a previously used syringe												
No	157	51.14 %	83	52.87	32	20.38	1		42	26.75	**1**	
Yes	150	48.86 %	97	64.67	26	17.33	1.09	0.94, 1.26	27	18	**1.18**	**1.01, 1.38**
Recently injected drugs (past 3 mo)												
No	138	35.94 %	62	44.93	23	16.67	1		53	38.41	1	
Yes	246	64.04 %	142	57.72	45	18.29	1.04	0.89, 1.21	59	23.98	**1.31**	**1.08, 1.59**
Recently shared previously use syringe (past 3 mo) (n = 150)												
No	108	72.00 %	71	65.74	18	16.67	1		19	17.59	1	
Yes	42	28.00 %	26	61.9	8	19.05	0.96	0.44, 1.19	8	19.05	0.97	0.78, 1.20
Substance use disorder treatment												
Prescribed MOUD ever												
No	268	69.79 %	116	43.28	43	16.04	1		109	40.67	**1**	
Yes	116	30.21 %	88	75.86	25	21.55	1.07	0.93, 1.22	3	2.59	**1.88**	**1.64, 2.14**
Recent (past 3 mo.) or currently on MOUD												
No	336	75.59 %	170	50.6	55	16.37	1		111	33.04	**1**	
Yes	48	24.41 %	31	70.83	13	27.08	0.96	0.79, 1.16	1	2.08	**1.61**	**1.44, 1.79**
Received treatment for any substance use disorder ever												
No	89	23.18 %	35	39.33	16	19.98	1		38	42.70	**1**	
Yes	295	76.82 %	169	57.29	52	17.63	1.11	0.91, 1.36	74	25.08	**1.45**	**1.13, 1.87**
Recent (past 3 mo.) or currently in treatment for any substance use disorder												
No	223	75.59	122	54.71	36	14.14	1		65	29.15	**1**	
Yes	72	24.41	47	65.28	16	22.22	0.97	0.82, 1.41	9	12.5	**1.29**	**1.10, 1.50**

### 3.1 Bivariate associations

Table 1 also presents the prevalence of each pattern of polydrug use involving methamphetamine (Simultaneous, Sequential, and Independent) and examines differences in demographic, socioeconomic, physical and mental health, and drug use behaviors in the three groups. Just over half of participants (53.1 %) reported simultaneous use, 17.7 % reported sequential use, and 29.2 % reported independent use.

Prevalence ratios (PR) describe the associations between the use patterns and the proportion reporting each exposure variable, while accounting for the reference group. This provides a measure of relative difference in prevalence between the exposure groups (for example between those with a history of incarceration vs. no history of incarceration) for each use pattern. Geographic differences were notable: simultaneous use was more prevalent in NM (66.5 %) compared to NV (39.9 %). No statistically significant associations were observed by gender or educational attainment.

Multiple indicators of poor socioeconomic conditions were significantly associated with use patterns. Among those who reported not having enough money to meet basic expenses, 58.4 % were individuals who used simultaneously compared to only 33.3 % among those living comfortably (PR comparing Simultaneous to Sequential: 1.33, 95 % CI 0.99, 1.80). Simultaneous use more prevalent among those who “often” ran out of food before they had money to buy more (59.5 %) compared to those who “never” worried (40.78 %; PR comparing Simultaneous to Sequential: 1.31, 95 % CI 1.07, 1.61; PR comparing Simultaneous to Independent = 1.27, 95 % CI 1.0, 1.6). Recent homelessness and recent incarceration were significantly associated with higher prevalence of simultaneous use relative to sequential and independent use. Despite high overall health insurance coverage (92.63 %), people engaged in simultaneous were 25 % less likely to report a care provider visit (44.89 %) compared to people using independently (36.93 %; PR comparing Simultaneous to Independent = 0.75, 95 % CI 0.63, 0.90). Several indicators of mental health were significantly associated with use patterns: those who reported having ≥ 1 mental health concern were significantly more likely to be engaged in simultaneous use (vs. sequential; PR = 1.70, 95 % CI 1.09, 2.64). However, individuals taking prescribed mental health medications were significantly less likely than their unmedicated counterparts to be engaged in simultaneous use (vs. sequential; PR = 0.70,95 % CI 0.51, 0.95). Inversely, participants who endorsed taking non-prescribed medications for mental health were more likely to use simultaneously than those who use independently.

The observation of lower simultaneous use among participants with multiple positive indicators of healthcare engagement, i.e. recent provider visit, taking prescribed medication, and engaged in therapy for mental health, prompted examination of the associations between these variables. A significantly higher proportion of those who reported a recent (last 3 months) health provider visit (vs no recent visit), reported being engaged in therapy for mental health (22.16 % vs. 7.69 %, respectively, X^2^: 16.3; p < 0.0001) and taking medications prescribed for mental health (32.31 % vs. 10.66 %, respectively; X^2^; p < 0.001). Participants who reported being prescribed stimulants during childhood had a 15 % higher prevalence of simultaneous use compared to sequential use (PR = 1.15, 95 % CI 1.0, 1.32). Trauma history showed strong associations with use patterns: those with lifetime trauma, recent trauma, and childhood trauma had significantly higher rates of simultaneous use (56.06 %, 58.27 %, and 55.97 %, respectively) compared to those without trauma history (Table 2).

**Table 2:** Multivariable models showing factors independently associated with Simultaneous use of methamphetamine and opioid compared to Sequential use and Independent use. (N = 386).

	Adjusted Prevalence Ratios^1^: Simultaneous use vs. Sequential use of methamphetamine and opioids		Adjusted Prevalence Ratios^2^: Simultaneous use of methamphetamine and opioids vs. Independent use (methamphetamine & no-opioid)	
Location	Adjusted PR	95 % CI	Adjusted PR	95 % CI
State: NM vs NV	1.07	0.94, 1.22	**1.68**	**1.41, 2.00**
Sociodemographic factors				
Homeless in the past 3 months − yes	**1.21**	**1.01, 1.44**		
Incarceration in the past 3 months − yes	**1.14**	**1.01, 1.30**	**1.23**	**1.04, 1.44**
Household financial situation				
Lives comfortably			1	
Meets basic expenses with a little leftover			**1.53**	**1.08, 2.17**
Don't have enough to meet basic expenses			**1.55**	**1.11, 2.15**
Health and mental health				
Has ≥ 1 mental health concern(s)	**1.59**	**1.07, 2.34**	1.36	0.94, 1.98
Currently taking Rx MH meds	**0.66**	**0.48, 0.91**	**0.68**	**0.49, 0.95**
Recent health provider visit	0.94	0.82, 1.07	**0.82**	**0.70, 0.97**

1 “State” retained in the model to control for confounding; “Recent health provider visit” retained due to clinical significance (p-value = 0.32).

2 “Has ≥ 1 mental health concern(s)” retained due to clinical significance (p-value = 0.10.).

Bold type indicates p-value ≤ 0.05.

Among those who endorsed transitioning from heroin to fentanyl, 82.9 % reported simultaneous use. And over half (58.63 %) of participants who had ever injected drugs engaged in simultaneous use, compared to only 31.2 % of non-injectors, and recent injection drug use (vs. no recent injection use) was associated with 31 % higher risk of simultaneous use (vs. independent use). Syringe sharing was more common among people engaged in simultaneous use (64.7 %) compared to other groups. Any history of substance use disorder (SUD) treatment also showed differences: among those with a history of taking medication for opioid use disorder (MOUD) simultaneous use was significantly higher (75.9 %) compared to those without MOUD history (43.3 %). Recent MOUD treatment showed similar patterns: 70.8 % of those with who reported receiving MOUD in the past 3 months used simultaneously compared to 50.6 % of those who had not received MOUD recently. Overall, any SUD treatment was associated with higher rates of simultaneous use (57.3 % vs 39.3 % for those without SUD treatment history).

### 3.2 Multivariable results

Factors found to be independently associated with polydrug use patterns involving methamphetamine are shown in Table 2. In the model comparing simultaneous use to sequential use, several factors were associated with higher prevalence of simultaneous use: homelessness (aPR = 1.21, CI: 1.01, 1.44), having mental health concerns (aPR = 1.5, CI: 1.07, 2.34), and recent incarceration (aPR = 1.14, CI: 1.01, 1.3). Currently taking prescribed mental health medications was associated with lower prevalence of simultaneous use (aPR = 0.66, CI: 0.48, 0.91). In the model comparing simultaneous use to independent use, financial hardship (not having enough money to meet basic expenses) was associated with 55 % higher prevalence of simultaneous use (aPR = 1.55, 95 % CI: 1.11, 2.17). Recent incarceration was also associated with higher prevalence of simultaneous use (aPR = 1.21, 95 % CI: 1.03, 1.42). Protective factors included taking prescribed mental health medications (aPR = 0.71, 95 % CI: 0.51, 0.99) and having recent healthcare visits (aPR = 0.85, 95 % CI: 0.72, 0.99).

## 4 Discussion

**Fig. 2:**
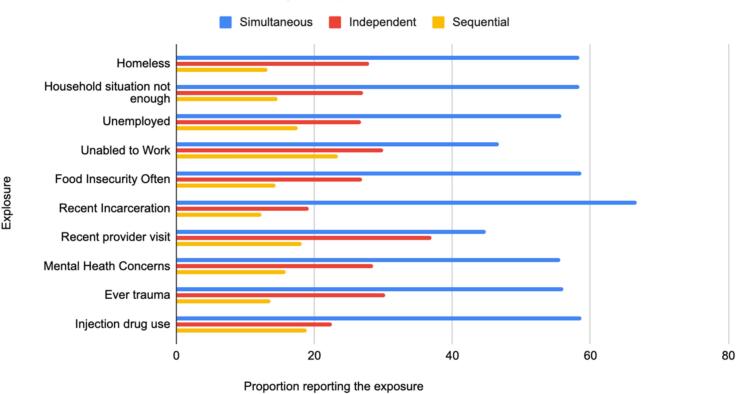
Prevalence of selected social, economic, carceral, health and drug related risk factors in co-users by use pattern.

In this study of methamphetamine and opioid co-use patterns, we found that simultaneous use (i.e., goofball use) was the most prevalent pattern, with just over half (53.13 %) of participants reporting this practice compared 17.71 sequential and 29.17 independent use. Simultaneous use was consistently associated with significantly higher prevalence of vulnerability indicators across multiple domains compared to both sequential and independent use. These domains included social factors (housing, incarceration), economic (food security, employment), health outcomes (mental health, trauma), and drug use risk behaviors (Fig. 2). In multivariable models adjusting for potential confounders, many of these indicators were independently associated with higher risk for simultaneous use. While bivariate analyses suggested that people who used sequentially had lower prevalence of some risk factors compared to those who used independently (for example 13.21 % vs. 27.92 % for recent homelessness), we did not directly compare these two groups in multivariable models, and the pattern varied across different outcomes. Relative to those who used sequentially, those who use simultaneously demonstrated significantly higher prevalence of food insecurity, past trauma, and recent incarceration. While simultaneous use was also associated with a higher crude prevalence of injection drug use and syringe sharing compared to sequential use, these differences were not statistically significant in bivariate or multivariable analyses. Social determinants of health are well established contributors to health in general, and are well established predictors and consequences of substance use (Lin et al., 2024).These findings highlight the particular importance of addressing socioeconomic vulnerabilities among PWUD engaged in polydrug use involving methamphetamine and opioids, and attending to the differential impacts of polydrug use patterns.

### 4.1 Health Risks Domain

Mental health concerns emerged as a critical factor differentiating use patterns. Almost all (90.4 %) respondents reported at least one mental health concern, and those who used simultaneously showed significantly higher prevalence of these. The strong association between taking non-prescribed medications for mental health and simultaneous use suggests self-medicating behaviors, aligning with national estimates that approximately one-quarter of individuals with mood disorders use substances to relieve symptoms (Bolton et al., 2009). Trauma exposure, both recent and historical, showed particularly strong associations with simultaneous use, consistent with literature linking trauma to polydrug use and stimulant use disorder (Clark et al., 2023, Ou et al., 2023). Early life experiences also appeared influential: the association between being prescribed stimulants as a child and simultaneous use suggests early risk factors, particularly relevant given the common prescription of amphetamines for ADHD and links to methamphetamine-associated psychosis in adulthood (Salo et al., 2013). Importantly, engagement with healthcare services emerged as a potential protective factor. Those receiving mental health treatment and prescribed medications showed lower risk of simultaneous use, suggesting that healthcare engagement might facilitate transitions to lower risk use patterns. These findings underscore the importance of integrated healthcare approaches that address both substance use and mental health needs.

Beyond these behavioral and clinical associations, emerging neuroimaging evidence provides insight into potential biological mechanisms. Neuroimaging studies have demonstrated that methamphetamine use is associated with structural and functional brain alterations, including reduced grey matter volume in frontal and limbic regions, white matter microstructural changes, and disrupted functional connectivity across reward processing and cognitive control networks (Liu et al., 2025). These neurobiological changes may represent mechanistic pathways through which methamphetamine exposure increases vulnerability to the compounded risks observed among those who use simultaneously, particularly through compromised decision-making, emotional regulation, and cognitive control capacities.

### 4.2 Treatment Challenges Domain

Our findings regarding treatment engagement reveal complex patterns with important implications for intervention development. While one in four respondents reported recent SUD or MOUD treatment, these interventions were associated with higher rates of simultaneous use relative to independent use. This seemingly paradoxical pattern may reflect that individuals with more severe use patterns are more likely to seek treatment, or that there is a need for interventions that recognize the goals other than abstinence, particularly given the complex interplay of trauma, mental health, and socioeconomic challenges we observed. The associations may also reflect the timing of our data collection (September 2022-August 2023), which coincided with efforts to expand access to opioid treatment nationally (Pessar et al., 2021).

### 4.3 Public Health Response.

The finding that simultaneous use is consistently associated with elevated vulnerability has significant implications for public health response strategies. Our classification approach, which clearly identifies simultaneous, independent, and sequential use patters, offers practical advantages for targeting interventions compared to more complex statistical methods. While Latent Class Modeling (LCM) (Karamouzian et al., 2022) provides sophisticated probability estimates, our straightforward behavioral classification more readily translates to harm reduction and clinical settings. Geographic variations between NV and NM suggest the need for locally tailored responses that consider regional patterns and resources. Both individual and community harm reduction approaches should adapt to address the distinct needs associated with different use patterns, particularly the elevated vulnerability observed among people who use methamphetamine and opioids simultaneously, especially given the continuously and rapidly changing drug supply. The documented shifts from injecting to smoking among many people who use drugs (Kral et al., 2021), further emphasize the need for adaptive harm reduction strategies that recognize and response to evolving use patterns.

### 4.4 Community Impacts.

Our findings have important implications for community resource allocation and support services. Simultaneous use was associated with significantly higher prevalence of homelessness, food insecurity, and recent incarceration compared to both other use pattern groups, suggesting this population requires more intensive community support. Both sequential and independent use was associated with lower risk profiles relative to simultaneous use, though the specific patterns varied by outcome, they may offer insights into successful community integration and support utilization. These findings emphasize the importance of considering social and economic contexts in developing responses to co-use patterns. Resource allocation should reflect that different polydrug use patterns are associated with different levels of vulnerability, with simultaneous use requiring particular attention, rather than focusing solely on opioid-specific interventions. Understanding these patterns can help communities develop more targeted and effective support systems.

## 5 Future Research and Limitations

Several methodological factors affect interpretation of our findings. The cross-sectional design prevents causal inference about transitions between use patterns or determination of temporal relationships between risk factors and use behaviors. While our two-state Mountain West sampling frame captured important geographic variations, convenience sampling limits generalizability to other regions or populations. Self-reported data may be subject to recall and social desirability bias, though non-differential underreporting would bias estimates toward the null. Our exclusion of legal substances like alcohol and tobacco, may limit comprehensive understanding or polysubstance use patterns. However, the consistency of associations across multiple indicators and the robust prevalence ratios suggest that these findings represent meaningful patterns despite these limitations.

Future research should include direct comparisons between sequential and independent use patterns in multivariable models to better understand whether these patterns differ meaningfully in terms of risk. Longitudinal studies are needed to understand transitions between use patterns and associated changes in vulnerability, particularly factors that might facilitate movement from higher to lower-risk patterns. The complex interplay between mental health, trauma history, and use patterns warrants particular attention, especially regarding how these factors influence vulnerability and transitions between use patterns. Evaluation of targeted interventions aimed at those using simultaneously could inform development of more effective treatment approaches. Investigation of regional variations could help identify structural, environmental, and policy factors that influence use.

## 6 Conclusions

These findings suggest the need for more comprehensive approaches that address both substance use patterns and broader social and economic contexts in which they occur, with particular attention to developing and evaluating interventions specifically engaged in simultaneous use. Both individual and community harm reduction approaches must adapt to address varying risk levels and the distinct challenges of polydrug use involving methamphetamine and opioids. Polydrug use is the norm (Jones et al., 2022). Public health messaging (Stalgaitis et al., 2023), and resource allocation (Combating the Opioid Crisis: Smarter Spending to Enhance the Federal Response, n.d.) should reflect that polydrug use exists along a spectrum of risk, rather than focusing solely on opioids. Surveillance systems, including tracking of drug supply changes, may benefit from adaptations to capture time-sensitive data collection on changing use patterns and associated risk levels (Canning et al., 2021, Delaney et al., 2023). Most importantly, harm reduction-oriented interventions that support transitions away from simultaneous use may be more acceptable and achievable for many PWUD, while still significantly reducing individual and community impacts of polydrug use. These findings suggest the need for comprehensive public health and clinical approaches that address both substance use patterns and the broader social and economic contexts in which they occur.

## CRediT authorship contribution statement

**Kimberly Page:** Writing – original draft, Supervision, Resources, Project administration, Methodology, Investigation, Funding acquisition, Formal analysis, Data curation, Conceptualization. **Mia Rae Kirk:** Writing – review & editing, Methodology, Investigation, Conceptualization. **Tristin Garcia:** Writing – review & editing, Resources, Project administration, Methodology, Investigation. **Haley Etchart:** Writing – review & editing, Resources, Project administration, Methodology, Investigation. **Benjamin Chase:** Writing – review & editing, Software, Methodology, Formal analysis, Data curation. **Robert W. Harding:** Writing – review & editing, Project administration, Methodology, Investigation, Data curation. **Jess Anderson:** Writing – review & editing, Supervision, Resources, Methodology, Formal analysis, Data curation. **May McCarthy:** Writing – review & editing, Methodology, Investigation. **Phillip Fiuty:** Writing – review & editing, Supervision, Project administration, Methodology, Investigation, Conceptualization. **Kathleen Reich:** Writing – review & editing, Supervision, Resources, Project administration, Methodology, Investigation. **Kelly Mytinger:** Writing – review & editing, Supervision, Project administration, Methodology, Investigation. **Olufemi Erinoso:** Writing – review & editing, Methodology, Investigation, Conceptualization. **Karla D. Wagner:** Writing – review & editing, Writing – original draft, Supervision, Project administration, Methodology, Investigation, Funding acquisition, Formal analysis, Data curation, Conceptualization.

## Declaration of competing interest

The authors declare that they have no known competing financial interests or personal relationships that could have appeared to influence the work reported in this paper.

## Data Availability

Data will be made available on request.
